# 

**Published:** 1868-09-01

**Authors:** 


### *CONTENTS:*

ORIGINAL PAPERS:

The Pathological Anatomy and Pathogenesis of Disseminated Chronic Pneumonia, and of Pulmonary Tubercles. Translated by Walter Hay, M.D., Associate Editor, Chicago.... ...... 547

Retroversion of the Pregnant Womb.......................... 559

Impacted Fracture of the Neck of the Femur, etc.................. 567

CORRESPONDENCE:

Homœopathy v. Common Sense.......... ................... 567

A Case of Monstrosity.............................................. 568

Letter from B. F. Lightfoot, M. D., Murrayville, Ill.. .. ......... 571

Philadelphia Correspondence......................................... 572

BOOK NOTICES:

A Theoretical and Practical Treatise on Midwifery, etc............ 573

The Anatomy and Histology of the Human Eye ................ 574

Klinik der Ohrenkrankheiten.......... ...................... 575

Progressive Locomotor Ataxia ............ .................... 575

Chicago—Past, Present and Future ............................. 575

EDITORIAL....... ........... ........... ................. 576

FOREIGN NOTES AND EXCERPTA.................... ............... 579

